# Ordered Aggregates of Fmoc-Diphenylalanine at Alkaline pH as a Precursor of Fibril Formation and Peptide Gelation

**DOI:** 10.1021/acs.jpcb.4c06796

**Published:** 2024-12-23

**Authors:** Emily Hughes, Nichole S. O’Neill, Reinhard Schweitzer-Stenner

**Affiliations:** Department of Chemistry, Drexel University, Philadelphia, Pennsylvania 19104, United States

## Abstract

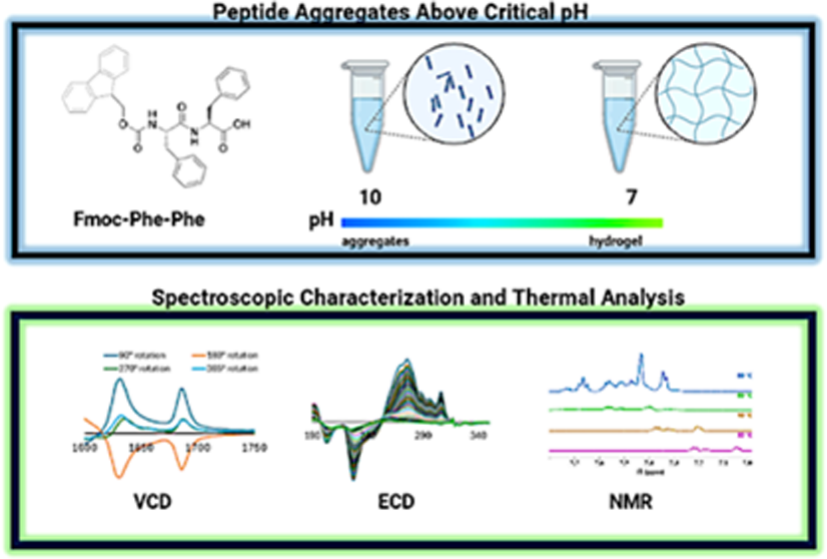

The ultrashort peptide *N*-fluorenylmethoxycarbonyl-phenylalanyl-phenylalanine (FmocFF) has been largely investigated due to its ability to self-assemble into fibrils (100 nm−μm scale) that can form a sample-spanning gel network. The initiation of the gelation process requires either a solvent switch (water added to dimethyl sulfoxide) or a pH-switch (alkaline to neutral) protocol, both of which ensure the solubility of the peptide as a necessary step preceding gelation. While the respective gel phases are well understood in structural and material characteristics terms the pregelation conditions are known to a lesser extent. The question we asked is to what extent the gel-forming fibrils are already partially formed, i.e., oligomers or protofibrils. Focusing on the pregelation conditions for the pH-switch method, we investigated the self-assembly of soluble FmocFF aggregates in alkaline pH by UV circular dichroism, IR, vibrational circular dichroism, and ^1^H NMR spectroscopy for different peptide concentrations and more systematically as a function of temperature. The temperature dependence of the UVCD spectra of FmocFF in H_2_O and D_2_O revealed a complicated isotope effect that affects the peptide backbone and fluorene conformations in peptide aggregates differently. Moreover, we found that the melting of formed aggregates depends on peptide concentration in a nonmonotonic way. At 20 mM the UVCD data revealed the population of at least two different thermodynamic intermediate states, which seem to differ in terms of the relative arrangement of the fluorene moiety. The IR spectrum of this sample at room temperature indicates an antiparallel β-sheet arrangement, as suggested earlier in the literature. However, we show that this interpretation can only be valid if one invokes a nondispersive redshift of the two amide I’ bands in a locally crystalline environment. The respective vibrational circular dichroism spectrum of the amide I’ region is consistent with a left-handed helically twisted structure of the formed aggregates. A comparison of our data with spectra of the aqueous gel phase suggests that fibrils in the latter resemble the ones at alkaline pH probed by our experiments.

## Introduction

It is now well established that polypeptides and proteins can self-assemble into supramolecular structures of considerable size (nm−μm) once their concentration exceeds the respective solubility limit.^1−7^ This notion in particular applies to intrinsically disordered systems, which lack any stable secondary structure. Prominent examples are Aβ_1–40(42)_, α-synuclein, and tau-protein, which in addition to natural functions are all implicated in some neurological diseases such as Alzheimer’s and Parkinson’s.^8−14^ The self-assembly of polypeptide chains is often facilitated in aqueous solutions because water is a poor solvent. It involves a plethora of noncovalent interactions such as hydrogen bonding (mostly but not exclusively between functional groups of the peptide linkages), van der Waals interactions between hydrophobic groups, ionic interactions between oppositely charged side chains of amino acid residues, and π–π-stacking between aromatic groups.^1,7,15−19^ While the individual strength of these contacts is generally moderate or even weak, their rather large number and cooperative effects lead to a considerable degree of stabilization.

Aromatic amino residues are the key promotors of the self-assembly of the so-called ultrashort peptides, which typically contain two or three residues.^5−7,19^ The most effective of the four natural aromatic amino acids is phenylalanine. Its presence in the islet amyloid polypeptide fragment NFGAIL and in the Aβ_16–22_ fragment of amyloid β-peptide enables fibril formation.^20−22^ Gazit and co-workers were the first to show that diphenylalanine can self-assemble into nanotubes, spheres, and plates.^23−27^ These supramolecular structures were termed aromatic dipeptide nanostructures. Later it was shown that even a single phenylalanine amino acid exhibits a capability to self-assemble.^27^ While most of the respective supramolecular structures discussed above are built with stacked β-sheets, the unblocked tripeptide GFG forms exceptionally long fibrils that are crystalline. The unit cell belongs to the *P*2_1_2_1_2_1_ space group. It contains four monomers, which adopt an inverse polyproline II-like conformation, located in the normally forbidden lower right quadrant of the Ramachandran plot.^28^

The final supramolecular structure of ultrashort phenylalanine-based peptides can be modified by replacing the N- and C-terminal groups of, e.g., diphenylalanine with aliphatic or aromatic groups. Tubular structures can be obtained with noncharged Ac-FF-NH_2_^29^ where the substitution of the ammonium group by the *tert*-butyl carbonate group (Boc-FF) facilitates the formation of both, tubes and spheres.^30^ Alternatively, the use of fluorenylmethoxycarbonyl (Fmoc) as the N-terminal blocking group promotes the formation of a sample-spanning network of fibrils that leads to gelation.^31^ The final product of the self-assembly process can also be modified by replacing one of the two phenylalanines with other amino acids such as serine or proline.^19^

A great deal of literature has been dedicated to the gelation of FmocFF which can be achieved by different protocols. The peptide is easily dissolved in a (polar) organic solvent, such as dimethyl sulfoxide (DMSO), ethanol, acetone, and fluoroisopropanol where gelation can be triggered by the addition of water.^5,32,33^ The relationship between the critical mole fraction of water in binary mixtures with DMSO and the volume fraction of FmocFF can be described by a power law, i.e., the phase boundary is linear in a log–log plot.^34,35^ The kinetics of the gelation process depends on the peptide concentration with a measurable lag time for volume fractions below 2.5 × 10^–3^. The storage modulus is in the 10^4^ Pa range.^32,36^

Pure hydrogels of FmocFF can be obtained by adopting a switch protocol, where the pH of the aqueous solution of the peptide is first adjusted to a value above 10.0 in order to achieve dissolution with millimolar concentrations.^37^ In the second step, HCl is titrated to the sample. The response of the sample depends heavily on the peptide concentration. At submillimolar concentrations, the peptide remains soluble at all pH values down to 2. At millimolar concentrations, two phases exist with pH values of around 9.5 and 6, where the titration displays plateaus (i.e., the pH remains constant in spite of the addition of HCl). The first phase transition produces a very weak elastic gel with storage moduli in the 10^–1^ Pa range. At acidic pH the gel is replaced by precipitations and phase separation. A stabilization of the gel phase was achieved by the addition of a salt. These results suggest that the p*K*-value of the carboxylate group of the C-terminus increases upon the self-assembly of FmocFF, which facilitates self-assembly in the neutral and even alkaline regions.

The question arises to what extent the FmocFF and its derivatives are in the monomeric state when it is dissolved at conditions that do not promote gelation (i.e., in pure solvents such as DMSO and in water at alkaline pH). The identification and characterization of precursors are important for a thorough understanding of peptide self-assembly and gelation processes. Recently, Levine et al. used ^1^H NMR, vibrational spectroscopy, and dynamic light scattering to obtain evidence for the formation of disordered soluble oligomers and to a more limited extent of protofibrils of FmocFF in DMSO in the centimolar and submolar range of peptide concentration.^38^ For millimolar concentrations of FmocFF in alkaline solution, a weak amide I’ band at 1625 cm^–1^ in the IR spectrum seems to be indicative of a β-sheet formation.^37^ Transmission electron microscopy images exhibit a small number of fibrils. Adams and colleagues conducted a very thorough investigation of the alkaline state of a related peptide in which the fluorenylmethoxycarbonyl group was replaced by naphthalene (2NapFF), which can be expected to exhibit a behavior similar to that of FmocFF.^32^ They provided multiple lines of experimental evidence for peptide self-assembly to occur at a pH above 10.0. Moreover, they identified two phases, one in the submillimolar regime where the peptide forms spherical micelles and another one in the millimolar range where micelles are worm-like. The latter were proposed as a precursor of network formation followed by the gelation of the sample. The respective IR spectrum seems to indicate an underlying β-sheet character, in agreement with the findings for FmocFF.^39,40^

IR spectroscopy on monomeric and self-assembled peptides in the aqueous solution are frequently performed by replacing H_2_O with D_2_O.^41−43^ The latter option is also used for neutron scattering experiments. On the other side normal H_2_O is used for UVCD and fluorescence spectroscopy as well as for material characterization experiments.^44−46^ From a chemical point of view, one would be inclined to believe that substituting H_2_O with D_2_O would not matter a lot. However, multiple lines of evidence suggest that this notion is incorrect. Folded proteins and fibrils of proteins and peptides are often more stable in D_2_O. Intermolecular hydrogen bonding between D_2_O molecules is stronger. Such observations can be explained by the so-called isotope effect where the zero-point energies of hydrogen bonds are lower in D_2_O than in H_2_O.^47^ McAulay et al. investigated how the gel formation of various blocked diphenylalanine peptides including FmocFF is affected by the replacement of H_2_O by D_2_O in the pH-switch protocol.^48^ Small-angle X-ray scattering data suggest some differences between the size of nanotubes formed at high pH/pD. More pronounced differences were obtained for the respective gel structures. Hamley et al. investigated the formation of nanotubes with the peptide RFL_4_FR and found that respective CD spectra measured with the peptide in H_2_O and D_2_O suggest β-sheet structures for both solvents, but with stronger intermolecular hydrogen bonding in D_2_O.^49^

The current study is aimed at exploring the soluble aggregate of FmocFF at an alkaline pH with spectroscopic means. UV circular dichroism, which is normally employed as a tool to determine the secondary structure of peptides and proteins, is used to probe the status of the fluorene and phenyl moieties of the peptide. This investigation is facilitated by the fact that the three lowest electronically allowed transitions of fluorene between 240 and 310 nm do not overlap with allowed phenylalanine and peptide transitions, which complicate the interpretation of the spectral components at lower wavelengths.^40,50^ IR and vibrational circular dichroism spectra of the amide I’ region provide additional structural details. Motivated by the above-cited experiments, which suggest that peptide and protein aggregation might proceed differently in H_2_O and D_2_O, we measured the UVCD spectra of FmocFF in both solvents. The latter were recorded as a function of temperature to determine the thermal stability of formed aggregates. Our data set is complemented by ^1^H NMR experiments, which combined with the recorded UVCD and IR spectra, shed some light on a multistep melting process.

## Material and Methods

### Sample Preparation

*N*-Fluorenylmethoxycarbonyl-phenylalanyl-phenylalanine (FmocFF) was purchased from Bachem and used without further purification. FmocFF was added to either H_2_O or D_2_O. NaOH or NaOD (0.5 M) was added to the aqueous suspensions of FmocFF until a pH of approximately 11 or above was reached. Great care has been taken to avoid high pH values to avoid hydrolysis. The samples were vortexed and sonicated for 3 h to fully dissolve the modified peptide. Depending on the concentration and the target pH, a required volume of dilute hydrochloric acid (0.085 M) was then added dropwise while the solution was vortexed and sonicated until the target pH was reached. The recorded IR and UVCD spectra of the final solution suggested that the amount of hydrolyzed peptide was negligible (*vide infra*). Hydrolysis would lead to a substantial decrease of amide I’ and a concomitant increase of the band assignable to the COO^–^ asymmetric stretching mode, which has not been observed.

### Circular Dichroism

Ultraviolet circular dichroism spectra were recorded between 185 and 350 nm on a Jasco J-1200 spectropolarimeter purged with nitrogen. The samples were loaded into a 0.1 mm cell and sealed with silicone-based vacuum grease to prevent evaporation at elevated temperatures. Spectra were recorded at a scan speed of 500 nm/min, a data pitch of 0.5 nm, and a bandwidth of 1 nm. Three accumulations were averaged for each time interval and D.I.T. of 1 s. For each measurement, the sample was first incubated at different initial temperatures for different time periods to ensure that equilibrium was achieved.

### Vibrational Spectroscopy

Fourier transform infrared and vibrational circular dichroism (VCD) spectra were recorded in the spectral range of 4000–400 cm^–1^ with a chiral IR/VCD spectrometer from BioTools using a 121 μm calcium fluoride cell. The spectral resolution was 8 cm^–1^. The VCD signal is represented as an average of spectra taken with eight orientations of the cell to eliminate the combined influence of sample anisotropy and instrument-based birefringence (0, 90, 180, and 270° for each side of the cell). Raw data and the average spectra are shown in Figure S1.^51,52^ The spectra were processed in Matlab, where the baselines were subtracted.

### ^1^H NMR Spectroscopy

The ^1^H NMR spectra of FmocFF in D_2_O were measured with a 500 MHz Varian instrument with a 5 mm HCN triple resonance probe. A 20 mM sample of FmocFF was dissolved in deuterium oxide. The pH was adjusted to a pH of 10.0 (pD = 10.5) by the addition of NaOD. The v.6.1 Varian software was used for processing all spectra. The sample was set at a spin of 20 Hz. The spectra shown in this paper cover the spectral range of 6.6–8.4 (ppm). They were recorded at temperatures between 25 and 80 °C at intervals of 5 °C. The fid files were opened in MestReNova software, which was used for the Fourier transformations and phase correction. The spectra were then baseline corrected and aligned on the D_2_O peak by using MestReNova software.

## Results

This section is organized as follows. We first present and discuss the UVCD spectra of 10 mM FmocFF in H_2_O and D_2_O measured at an alkaline pH (pD) and room temperature. While UVCD spectra of FmocFF and other Fmoc-dipeptides have been reported in the literature (Figure S2), we are not aware of any assignment of observed Cotton bands to specific electronic transitions. This gap is filled in the first paragraph of this section. In the second paragraph, we present and analyze the temperature dependence of CD spectra of the above alkaline sample. The third paragraph provides a brief description of the concentration dependence of the UVCD spectra of the FmocFF sample in D_2_O. Our focus on deuterated water as a solvent is briefly justified. For reasons specified below the UVCD spectra of FmocFF are not an ideal tool for a secondary structure analysis which we present in the fourth paragraph based on the amide I’ region of the IR and VCD spectrum. This section concludes with a brief description of the ^1^H NMR spectra of FmocFF in D_2_O where we focus on the CH protons of the Fmoc moiety.

### Circular Dichroism Spectra at Room Temperature

**Figure 1:**
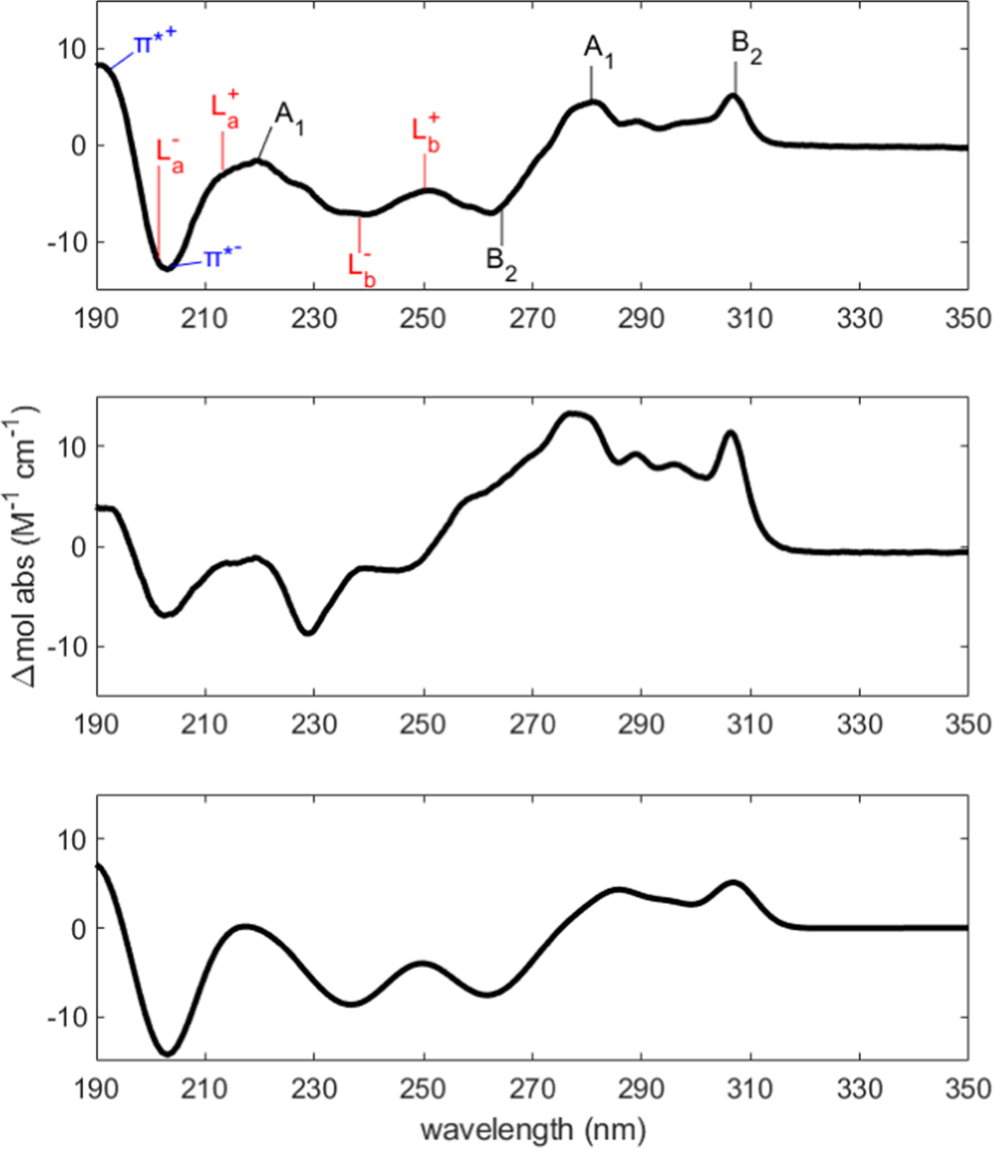
Upper panel: UVCD spectrum of 10 mM FmocFF in H_2_O recorded at 20 °C and a pH of 10.6. Electronic transitions indicated in the figure are discussed in the text. Middle panel: UVCD spectrum of 10 mM FmocFF in D_2_O recorded at 20 °C and a pH of 10.8. Lower panel: Simulated CD spectrum resembling the experimental spectrum in the upper panel calculated as a superposition of Gaussian Cotton bands, the parameters of which are listed in Table 1.

Figure 1 depicts the UVCD spectra of 10 mM FmocFF in H_2_O and D_2_O, respectively. The corresponding pH values were 10.59 (pD 11.1) and 10.80 (pD 11.3). While sharing some similarities, the two spectra are not identical, suggesting the occurrence of an isotope effect.^47^ The multitude of positive and negative maxima in the entire spectral region indicates that FmocFF is not monomeric under the above conditions. If it were monomeric the CD signals should be weak in the region above 250 nm, which is dominated by electronic transitions of the Fmoc group (*vide infra*), since the latter is not chiral. We assigned positive and negative maxima in the spectra to the electronic transition of three chromophores, i.e., the peptide groups (blue), the phenyl ring of the phenylalanine side chain (red), and the fluorene moiety of the Fmoc group (black). Electronic transitions of the fluorene in the gas and crystal phase have been reported by Nguyen et al.^40^ The corresponding absorption and Cotton bands cover nearly the entire region of the depicted spectra. The electronically allowed fluorene transitions are classified in terms of the irreducible representations A_1_ and B_2_ of the C_2v_ point group. The two transitions coincide with the long and short axes of the fluorene moiety, respectively. For phenylalanine, benzene is an appropriate reference system. We follow Giubertoni et al. in that we adopt the nomenclature of Platt and assign the couplet-like signal between 230 and 200 nm to excitonic coupling between L_a_ transitions.^47,53^ However, this couplet overlaps with a positive couplet assignable to peptide transitions (multiple π → π* and n → π* transitions mixed by configurational interactions^54,55^) the rather broad negative component of which in the region between 200 and 240 nm constitutes the baseline for the L_a_-couplet. In addition, a positive Cotton band assignable to an A_1_-transition of fluorene overlaps with the L_a_-couplet on the low-energy side. The weaker couplet in the region between 240 and 260 nm coincides with the position of the L_b_-band of the phenyl ring. The bands above 260 nm are assigned to fluorene transitions. The B_2_-band appears as a very sharp band in the absorption spectra of FmocFF gels where it has been assigned to an exciton peak.^45^ However, in our case the clearly visible vibronic structure on the high energy side argues against any significant excitonic character at our experimental conditions.^56^

To make the above assignment more plausible, we simulated the spectrum of FmocFF in H_2_O by superimposing assumed unnormalized Gaussian band profiles for each of the proposed spectral components. The simulation was carried out on a wavenumber scale since canonical band profiles are not symmetric on a wavelength scale. Amplitudes, wavenumber positions, and halfwidths were decided based on the experimental spectrum in Figure 1. The respective values are listed in Table 1. The simulated spectrum is displayed in Figure 1. It reproduces all the basic features of the experimental spectrum.

**Table 1:** Listing of Gaussian Band Parameters Used for the Simulation of the UVCD Spectrum Shown in the Lower Panel of Figure 1

band position (cm^–1^)	band position (nm)	halfwidth (cm^–1^)	peak value (M^–1^ cm^–1^)
52,631	190	1800	8
43,478	230	5400	–8
49,261	203	1800	–13
46,948	213	1800	5
44,843	223	1800	5
41,666	240	1800	–3
40,000	250	1800	5
38,167	262	1800	–7
35,842	279	1800	4
34,965	286	700	2
33,783	296	700	2
32,573	307	600	5

**Figure 2:**
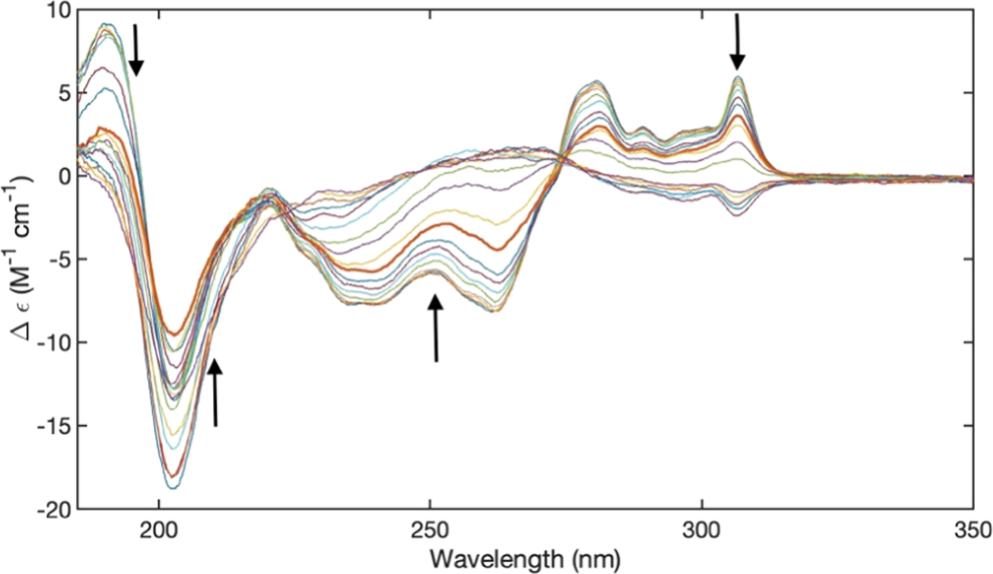
UVCD spectrum of 10 mM FmocFF in H_2_O measured at different temperatures at pH 10.6. The depicted spectra were recorded at 4, 6, 8, 10, 16, 10, 26, 30, 36, 40, 46, 50, 56, 60, 66, 70, 76, and 80 °C. The arrows indicate the direction of changes with increasing temperature.

While the overall structures of the spectra in Figures 1 and 2 are similar, the relative intensities of Cotton bands are distinctly dissimilar. The intensities of the positive Cotton bands of fluorene in D_2_O are nearly twice the ones in the spectrum of the molecule in H_2_O. The B_2_-transition at 260 nm seems now to be split into an excitonic couplet that is overlapped by the intense A_1_-band. It is possible that the positive band of this couplet is obscured by the A_1_-band in the spectrum of the H_2_O sample. While the CD signals associated with the two lowest electronic transitions of the Fmoc group are clearly enhanced, the very opposite seems to be the case for the L_a_-couplet. Taken together, these data indicate a different degree of self-assembly of FmocFF in H_2_O and D_2_O, with a more significant involvement of π–π-stacking in the latter.

**Figure 3:**
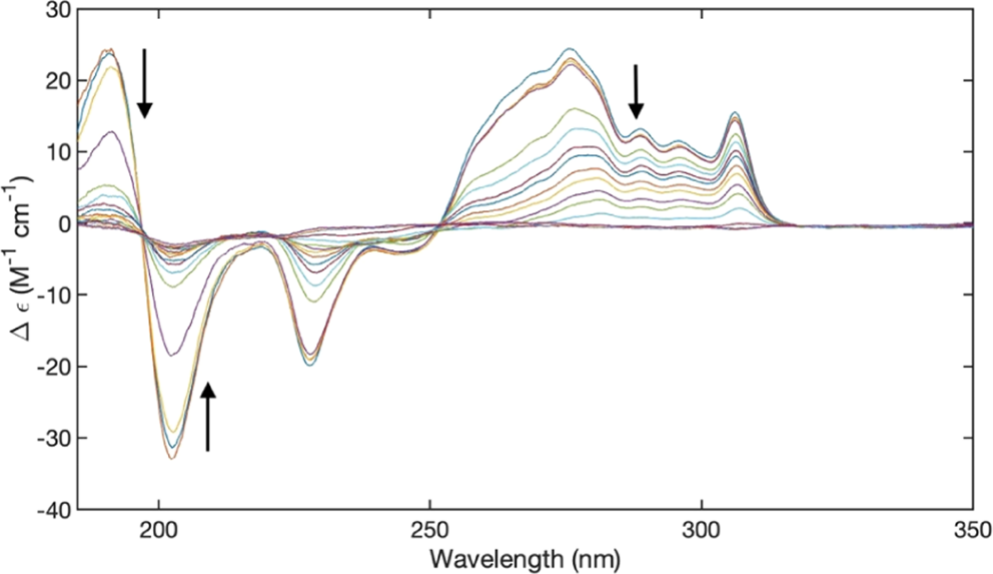
UVCD spectrum of 10 mM FmocFF in D_2_O measured at different temperatures at pH 10.8. The depicted spectra were recorded at 4, 6, 8, 10, 16, 10, 26, 30, 36, 40, 46, 50, 56, 60, 66, 70, 76, and 80 °C. The arrows indicate the direction of changes with increasing temperature.

Figures 2 and 3 show the UVCD spectra of alkaline FmocFF in H_2_O and D_2_O recorded at different temperatures. In both cases, the rotational strengths of involved transitions decrease with increasing temperature. Both sets of spectra exhibit a clear isodichroic point at 274 (H_2_O) and 252 nm (D_2_O). In the 220 nm region, the dichroism values are very similar, but there is no clearly identifiable isodichroic point. This observation suggests that the changes probed by the fluorene part of the CD spectrum predominantly involve conversion between only two states.

The peptide concentration of 10 mM chosen for the above measurements lies well above the second critical micelle concentration identified for 2NapFF by Adams and co-workers,^32^ so that one can expect a dominance of the worm-like state of peptide aggregates. We focus on this assumed state of peptide aggregates because it has been proposed to be the precursor state of gelation. However, we wondered whether the state of aggregation would still be concentration-dependent in this region. To address this point, we measured the temperature dependence of the UVCD spectrum of FmocFF for peptide concentrations of 5 and 20 mM. The former just lies slightly above the region of the second critical micelle concentration (1.4 mM for 2NapFF), while the latter is high enough to allow for further investigations by IR and VCD spectroscopy. For the sake of clarity, we focused on D_2_O as the solvent since it is required for the IR and VCD experiments to be described below. We also found UVCD spectra of D_2_O samples to be of better quality (i.e., less noisy).

**Figure 4:**
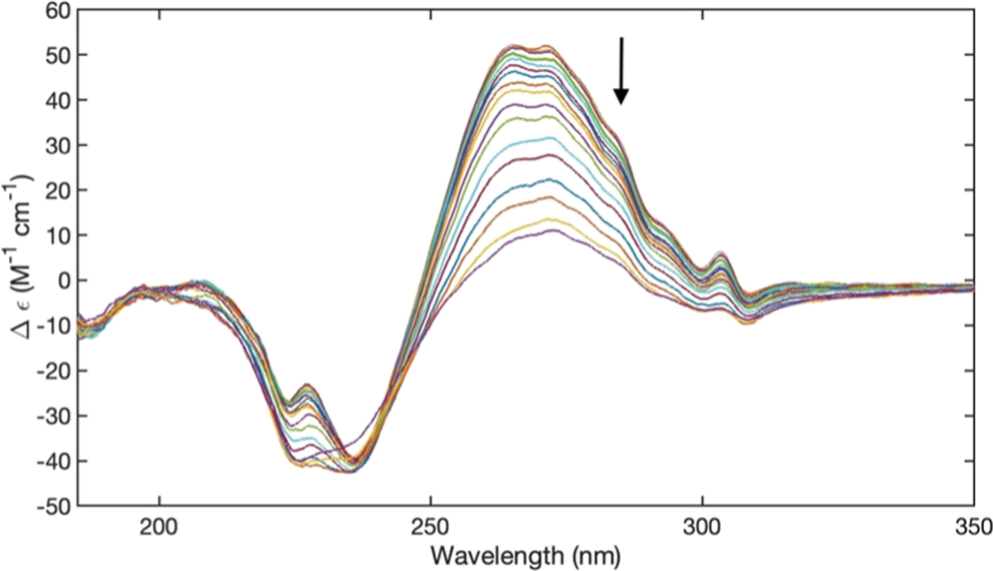
UVCD spectrum of 5 mM FmocFF in D_2_O measured at different temperatures at pH 10.6. The depicted spectra were recorded at 4, 6, 8, 10, 16, 10, 26, 30, 36, 40, 46, 50, 56, 60, 66, 70, 76, and 80 °C. The arrow indicates the direction of changes with increasing temperature.

**Figure 5:**
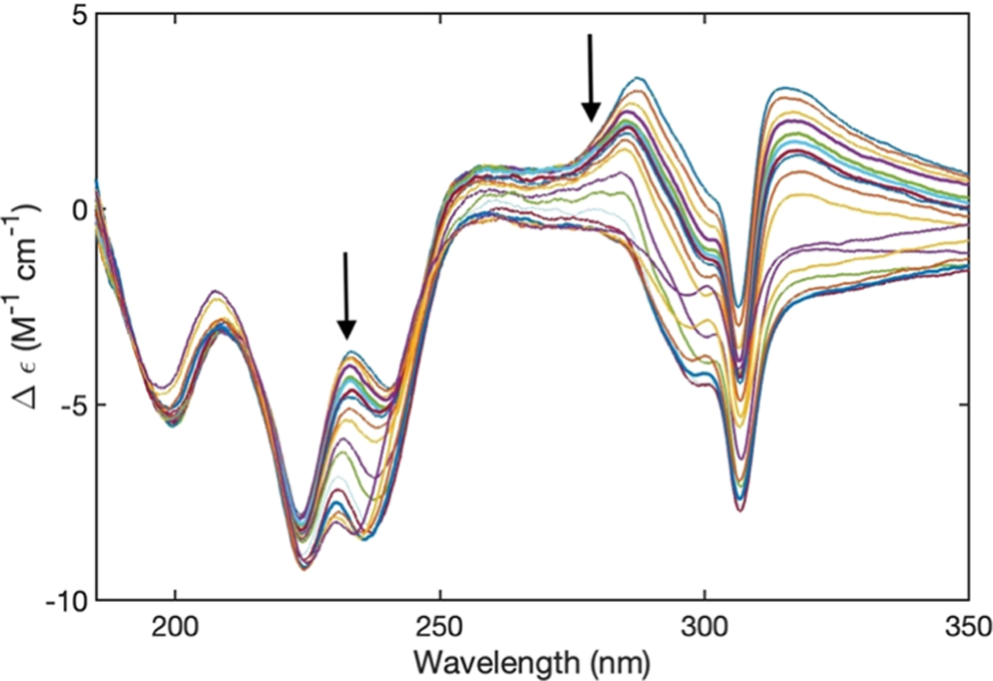
UVCD spectrum of 20 mM FmocFF in D_2_O measured at different temperatures at pH 10.6. The depicted spectra were recorded at 4, 6, 8, 10, 16, 10, 26, 30, 36, 40, 46, 50, 56, 60, 66, 70, 76, and 80 °C. The arrows indicate the direction of changes with increasing temperature.

Figures 4 and 5 depict the UVCD spectra of 5 and 20 mM FmocFF in D_2_O recorded at different temperatures, respectively. The spectrum measured at the lower concentration is much less structured than that observed with 10 mM (Figure 1). It looks like a slightly positively based negative couplet with very broad Cotton bands that exhibit some fine structure. In the fluorene region above 250 nm the dichroism values are all positive while they are negative below. The spectra seem to indicate an isodichroic point at 240 nm, but a closer inspection shows that not all spectra share the same data point within the experimental accuracy. The spectra recorded with the 20 mM sample are more structured, and the electronic transitions indicated in Figure 1 are identifiable. Additionally, a pronounced and very sharp negative maximum is now depicted at the B_2_-position at 306 nm. Interestingly, these spectra resemble to a significant extent those earlier reported for a FmocFF hydrogel (Figure S1),^57^ but they are more structured than the latter. The data seem to indicate that the chirality of the Fmoc chain in the peptide aggregates at 20 mM must be the opposite of that at 5 and 10 mM. We will analyze this spectrum in more detail in the Discussion section.

**Figure 6:**
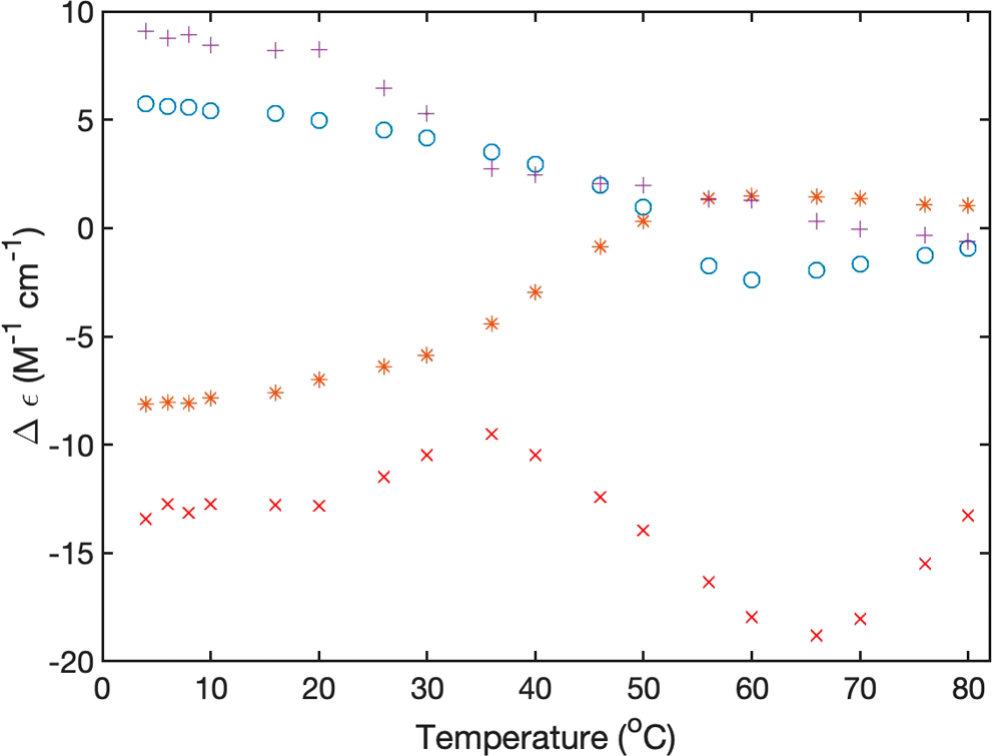
Temperature dependence of the dichroism values of 10 mM Fmoc FF in H_2_O at pH 10.6 recorded at 190 nm(+), 203 nm (×), 262 nm (*), and 306 nm (o).

**Figure 7:**
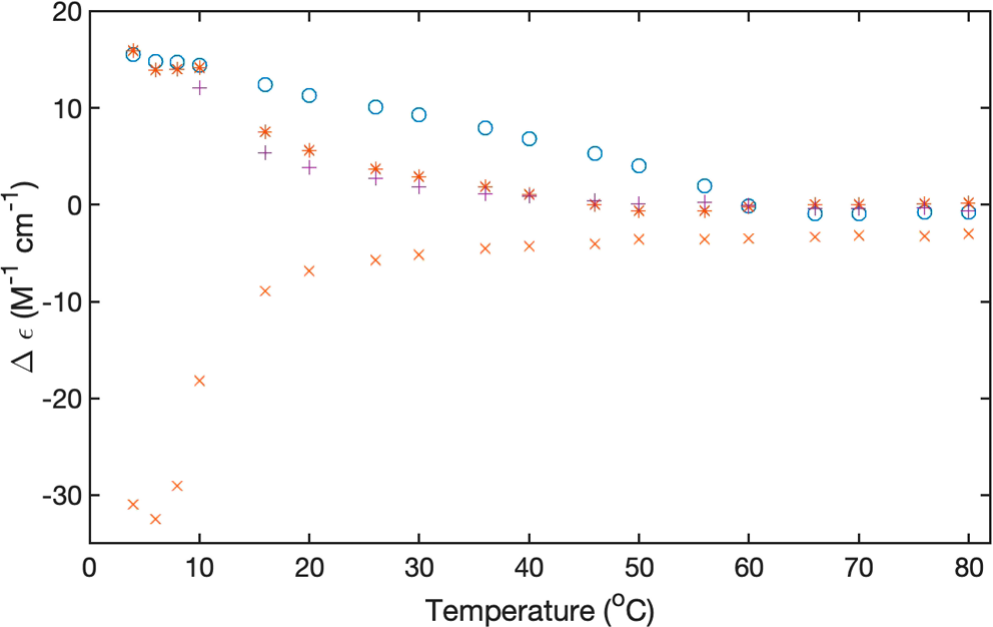
Temperature dependence of the dichroism values of 10 mM FmocFF in D_2_O at pH 10.8 recorded at 190 nm(+), 203 nm (×), 262 nm (*), and 306 nm (o).

The temperature dependence of the CD spectra deserves some further inspection. We first focus on differences between spectra of the 10 mM peptide solution in H_2_O and D_2_O. Figures 6 and 7 show plots of the dichroism values at 190 nm (peptide), 206 nm (phenylalanine and peptide), and 262 and 306 nm (fluorene) as a function of temperature. For FmocFF in H_2_O, the sigmoidal slope of the curve appears stretched. The 190 nm dichroism value of FmocFF also exhibits a stretched sigmoidal behavior, but the transition temperature seems to be somewhat lower. The 203 nm dichroism reveals a more complex and difficult-to-interpret situation. The data exhibit a relative maximum at 40 °C and a minimum (i.e., a negative maximum) at 65 °C, which indicates the existence of thermodynamic intermediates at these temperatures.

The behavior in D_2_O is qualitatively different (Figure 6). None of the data sets seem to indicate a leveling off at low temperatures. For 203, 262, and 306 nm, the melting curves look like very stretched sigmoidal curves from which only the upper part could actually be measured. The 190 nm data exhibit a much less stretched curve, and the corresponding melting temperature seems to be lower than the ones associated with the two aromatic groups. Overall, these data indicate that the thermal decomposition of FmocFF aggregates is more heterogeneous in D_2_O than in H_2_O. A semiquantitative analysis of some of the displayed data is presented in the Discussion section.

**Figure 8:**
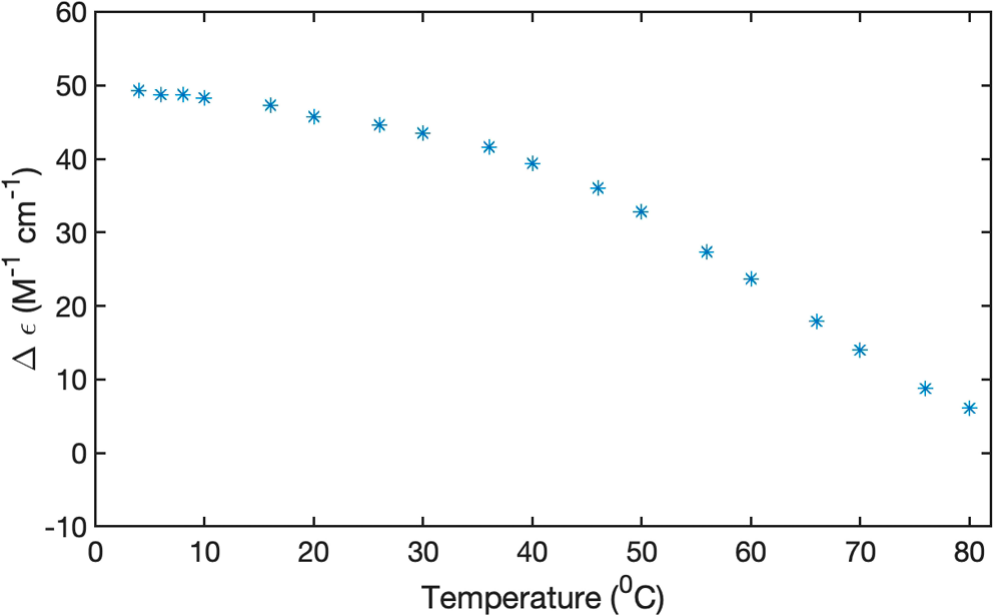
Temperature dependence of the dichroism values of 5 mM FmocFF in D_2_O at pH 10.6 recorded at 262 nm.

**Figure 9:**
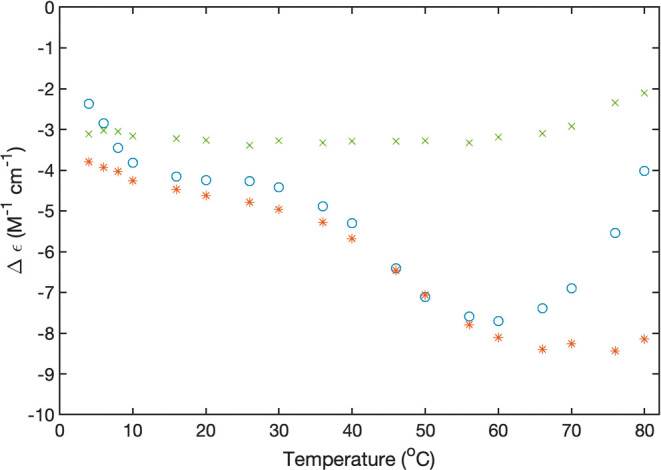
Temperature dependence of the dichroism values of 20 mM FmocFF in D_2_O at pH 10.6 recorded at 190 nm(+), 203 nm (×), 262 nm (*), and 306 nm (o).

Figures 8 and 9 display the CD temperature plots for 5 and 20 mM FmocFF in D_2_O. For the 5 mM data set, we plotted only the values at 235 nm. They exhibit a stretched sigmoidal behavior indicative of a transition temperature of around 50 °C. For 20 mM, we plotted the temperature dependence of the dichroism at 207 nm, 235 nm (both phenylalanine), and 306 nm (fluorene). They reveal a more complex behavior. The rotational strength at 207 nm starts to decrease only above ca. 65 °C. The dichroism values recorded at the other two wavelengths clearly suggest a thermodynamic intermediate at 60 °C in which the negative rotational strength is significantly enhanced. From there it decreases toward higher temperatures. These data suggest that the 20 mM FmocFF aggregates are by far more thermally stable than the ones formed at 5 and 10 mM.

### IR and VCD Spectroscopy

**Figure 10:**
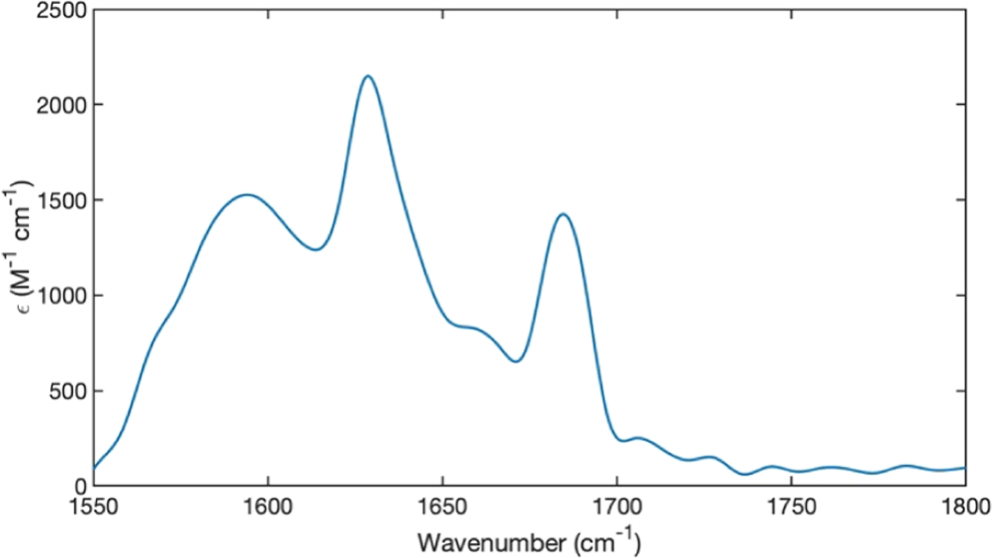
FTIR spectrum of the amide I’ region of 20 mM FmocFF at pH 10.0 in D_2_O. The presented spectrum has been calculated as an average over 8 spectra taken with the spectrometer cell at 0° (arbitrary), 90°, 180°, and 270° for both sides of the cell. The baseline was subtracted with a polynomial function constructed using Matlab.

Figure 10 depicts the amide I’ region of the IR and VCD spectrum of 20 mM FmocFF in D_2_O. The IR spectrum contains three discernible bands. A broad band at 1594 cm^–1^ is assignable to the antisymmetric COO^–^ stretching mode.^58^ Its unusual width indicates significant inhomogeneous broadening due to conformational heterogeneity. A similar observation has been made for fibrils of GHG and GFG^28,59^ in the gel phase. The bands at 1628 and 1663 cm^–1^ result from the C-terminal and N-terminal amide I’ band, respectively. Here, we follow our earlier practice to use the amide nomenclature for the latter, even though the C=O belongs strictly to the carbamate group.^38^ As a consequence, it appears always very much blue-shifted from the “pure” amide I position. Both bands are clearly red-shifted from the respective positions in a monomer spectrum (1648 cm^–1^ for a C-terminal amide I’ and 1688 cm^–1^ for the carbamate amide I’).^39^

The initially recorded VCD spectrum of the amide I’ region (Figure S1, denoted as R1) unexpectedly exhibited two rather intense positive Cotton bands. This differs from amide I’ VCD profiles of peptide oligomers and fibrils which generally appear as negatively biased couplets or even negative Cotton bands.^60−63^ We wondered whether the obtained VCD signals were affected by sample anisotropies, combined with instrumental birefringence (i.e., residual static birefringence caused by the photoelastic modulator and linear birefringence related to polarization imperfections of the detector). Such anomalies can produce rather strong VCD signals, which can obfuscate a less intense sample signal. One can be identified by rotating the cell of the IR/VCD spectrometer. Buffeteau, Merten, and their respective co-workers showed that linear birefringence and dichroism can be eliminated by averaging over all the spectra taken with rotated and flipped cells.^51,52^ Indeed we discovered that the magnitudes of the VCD maxima change when either an in-plane or out-of-plane rotation of the sample cell is performed. A similar observation has been made for the earlier investigated gel phases of GHG and GFG.^28,51,52,59^ In line with the above suggestions by Merten et al., we took eight measurements, i.e., an arbitrarily defined 0° and three measurements after consecutive 90° rotations on each side (Figure S1). The result in Figure 12 reveals considerable rotational strength for both amide I’ bands. A positively biased negative couplet was observed at the position of the C-terminal band. The corresponding carbamate group signal looks all positive, but it is more likely that the negative component overlaps by the wing of the positive Cotton band at low wavenumbers. Here, it should be noted that the measured profiles are actually envelopes of multiple excitonic states produced by coupling between the individual excited vibrational states of peptides and carbamate groups. Hence,^28,59^ the VCD signals are most likely produced by a left-handed chiral arrangement of β-sheet peptides in the formed aggregates.

### NMR Spectroscopy

**Figure 11:**
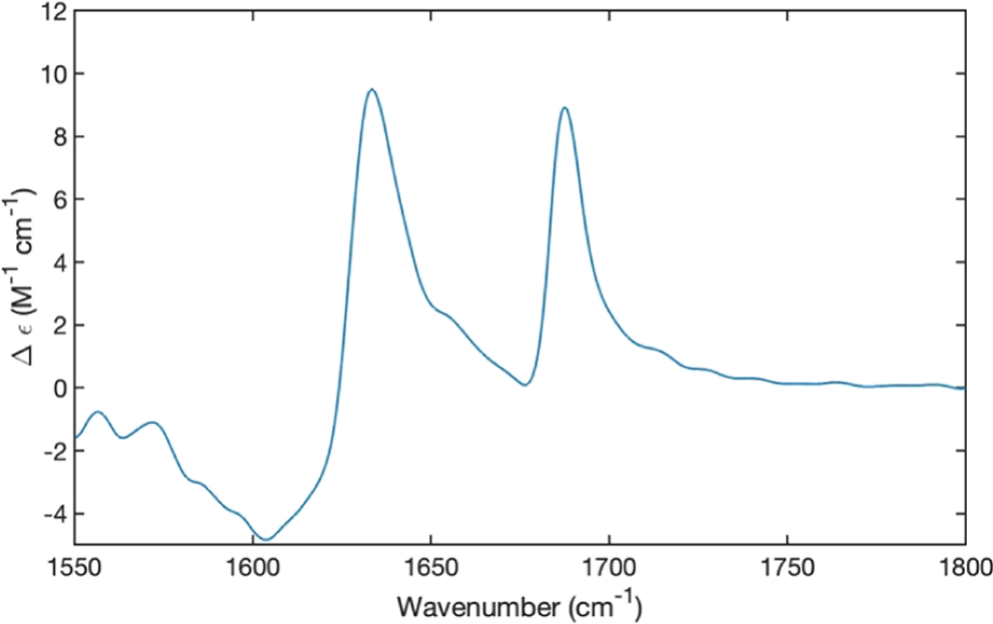
VCD spectrum of the amide I’ region of 20 mM FmocFF at pH 10.0 in D_2_O. The presented spectrum has been calculated as an average over 8 spectra taken with the spectrometer cell at 0° (arbitrary), 90°, 180°, and 270° for both sides of the cell. The baseline was subtracted with a polynomial function constructed using Matlab.

**Figure 12:**
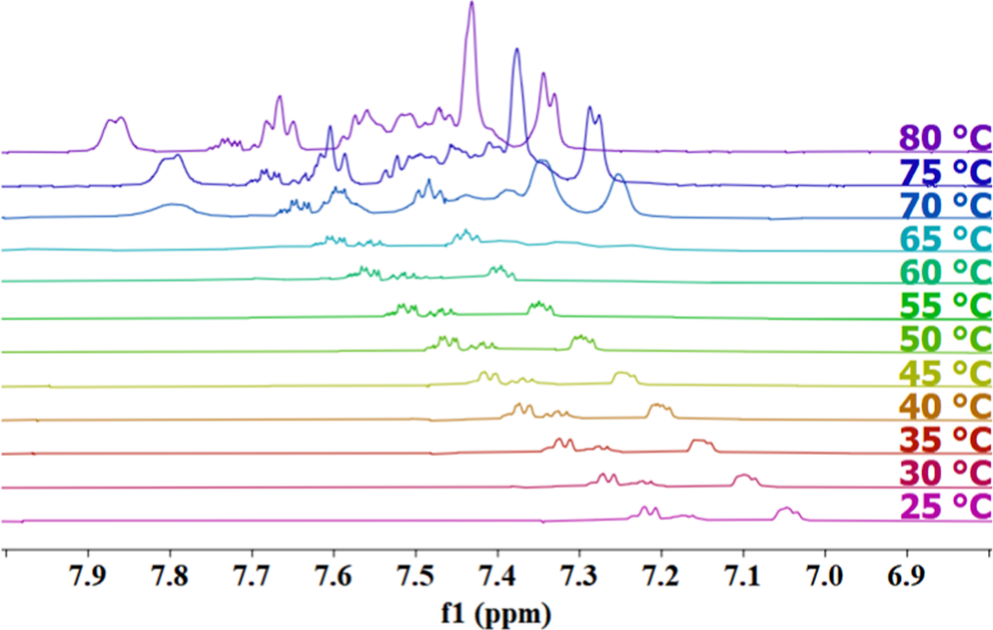
^1^H NMR spectrum of 20 mM FmocFF in D_2_O at pH 10.0 recorded at the indicated temperature. The depicted spectral region shows signals attributable to fluorene hydrogens.

Figure 12 exhibits the ^1^H spectrum of the Fmoc proton region of 20 mM FmocFF in D_2_O recorded at different temperatures. Up to 65 °C the signals are very weak, suggesting a significant degree of peptide aggregation. The signals themselves move all downfield, which can be understood as resulting from deshielding effects due to changes in the order of the fluorene arrangement.^64^ Above 65 °C the spectral lines become more intense, more numerous, and better resolved. This behavior correlates nicely with the reduced dichroism of the Fmoc moiety, which we inferred from the temperature plot in Figure 11. It thus strongly corroborates the notion that peptide aggregates formed at 20 mM concentration are very thermostable up to 65 °C while significant structural rearrangements already occur at lower temperatures.

## Discussion

### General Conclusions

The most general conclusions that can be drawn from our spectroscopic data are that FmocFF in alkaline solutions with a pH value above 10 self-assembles into rather ordered aggregates in the millimolar and centimolar region. The rather large rotational strength reflected by the UVCD spectra between 190 and 306 nm is indicative of chirality mostly induced by interactions between phenylalanyl residues and fluorene moieties, respectively. The formed aggregates melt at high temperatures, as probed by a drastically reduced rotational strength for electronic transitions assignable to the aromatic chromophores. The concentration dependence of the UVCD spectrum indicates a rather fluid ensemble of peptide aggregates in which the additional incorporation of peptide monomers at higher concentrations (20 mM in our case) leads to different relative arrangements of peptides with very different thermal stabilities. At 20 mM, FmocFF aggregates become thermally stable with a melting temperature above 60 °C. A comparison of CD spectra of FmocFF in H_2_O and D_2_O indicates an isotopic effect where the larger rotational strength obtained for the latter suggests a higher degree of aggregation and order. The notion of an ensemble of at least somewhat ordered FmocFF aggregates is corroborated by IR and VCD data which suggest an underlying β-sheet structure. The situation reflected by our experimental data is clearly distinct from the one of FmocFF in DMSO in the same concentration range where the peptide assembles more into amorphous aggregates.^38^ In what follows, we discuss some specific aspects of the obtained spectroscopic data.

### Interpretation of UVCD Spectra

An assignment of individual Cotton bands identifiable in the UVCD spectrum of 10 mM FmocFF in D_2_O has already been presented in the preceding section. Here, we comment on individual features in some more detail.

The positive couplet in the region between 185 and 210 nm of the 10 mM spectrum is predominantly due to excitonically coupled π → π* and n → π* transitions of the peptide groups.^54,65−68^ The corresponding negative maximum is additionally enhanced by a superposition with the negative maximum of the L_a_-state couplet of phenylalanine.^50^ For the simulation of the spectrum in Figure 1 we had to assume an asymmetric couplet for the peptide transition with a much broader negative maximum. Generally, such a CD signal is indicative of a β-sheet or of some type of right-handed helical structure.^69^ One might prefer the latter option because of the rather large amplitude of the negative Cotton band, but generally the CD spectra of, for example, right-handed α-helical or 3_10_-structures depict two negative maxima above 200 nm. On the other hand, the negative maximum suggested by our modeling is somewhat too pronounced compared with canonical β-sheet spectra. The corresponding spectrum of the 20 mM sample shows a less pronounced negative maximum just below 200 nm which is due to the reduced strength of the L_a_-couplet. The positive maximum is blue-shifted out of the spectral window. These data suggest some minor structural changes of the peptide backbone structure which are not qualitative in nature. We will discuss the backbone structure of FmocFF in more detail below when we analyze the IR and VCD spectra in Figure 10.

The aromatic fluorene and phenyl groups are not intrinsically chiral. Hence, they would not produce a CD signal. However, even in a monomer, electronic coupling between transitions of these groups and the chiral peptide backbone could induce some optical activity but not to the extent observed for FmocFF at room temperature. We should first note that the CD signals assignable to phenylalanine and the Fmoc group are qualitatively different. The L_a_ and L_b_ signals of the former exhibit a clear couplet character which is indicative of excitonic coupling between phenylalanine groups.^70^ In order to produce such a couplet, the arrangement of the phenylalanine groups in the formed aggregates must be chiral. The couplets of both transitions suggest that the chiral structure is left-handed. The dominance of excitonic coupling further suggests that the respective stacked phenylalanines in the aggregated state follow the Kasha theory for aromatic groups, which are stacked without producing significant orbital overlap,^56^ This would allow for some additional charge transfer transitions between adjacent groups. In a helical arrangement, CD signals can become quite intense owing to the long-range behavior of electronic circular dichroism which can be operative even if the distance between chromophores is large compared with their size.^71,72^

Based on our assignment shown in Figure 1 the CD activity of the fluorene group is quite different from the one of the phenylalanine’s. The two lowest energy transitions between 270 and 320 nm give rise to positive Cotton bands, which include vibronic transitions. The third (B_2_) transition seems to give rise to a weak couplet. The fourth transition (A_1_) produces a positive Cotton band that is overlapped by the broad negative band of the peptide CD.

If we assume that the rotational strength obeys the Rosenberg equation:
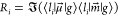
1where μ⃗ and *m⃗* label electronic and magnetic dipole operators, *g* represents the ground state, and *l*_*i*_ is the ith vibronic excited states. For isolated aromatic monomers, the magnetic transition dipole moment results from changes in ring currents in the molecular plane so that it is oriented perpendicular to in-plane electronic transitions. As a consequence, the corresponding rotational strength is zero. In the presence of excitonic coupling, a magnetic moment can be induced by its neighbors, which depends on the degree of excitonic mixing and the relative orientation of corresponding electronic transition dipole moments. This gives rise to a couplet.^70^ However, if stacked chromophores are close enough to allow for an overlap of π-orbitals interacting chromophores of oligomers must be considered as a whole system in which charge transfer transitions can occur between chromophores.^56^ In the asymmetric arrangement in a J-aggregate an intermolecular ring current could produce a magnetic transition dipole moment that is no longer perpendicular to the electronic dipole moment associated with excitonic and charge transfer transition. This could give rise to the asymmetric dichroism observed for three of the four fluorene transitions. We should emphasize in this context that in such a case electronic transitions can no longer be classified in terms of irreducible representations of the C_2v_-group. Computational modeling of helical chains of J-aggregates is beyond the scope of this paper. Here, we just conclude that the CD spectrum of the fluorene chromophore as measured with peptide concentrations of 5 and 10 mM indicates a close proximity of Fmoc groups in the formed aggregates.

Our data reveal a very pronounced concentration dependence of the CD spectrum, which is not linear. Hence, we do not just probe the growth of the formed aggregates without any structural change. The overall shapes of the spectra taken with 5 and 10 mM peptide concentrations are similar, but the fine structure of the latter indicates a more ordered structure which allows for the identification of individual components (*vide supra*). Apparently, the situation changes somewhat at 20 mM. While the CD signals of the L_a_ and L_b_ transitions still exhibit a couplet, a very sharp negative Cotton band appears at the position of the 306 nm B_2_-transition. As described in the Results section, this negative band gains intensity with increasing temperature before it reverses above 60 °C. Positive signals now appear on the high and low energy side of the negative maximum which become slightly negative in the intermediate state at 60 °C, most likely due to the overlap with the negative 307 nm band. Altogether the CD spectrum in the region between 280 and 320 nm looks like a superposition of two negatively biased couplets, one positive and the other one negative. Currently, we do not have a final explanation for these observations. We just hypothesize that the obtained CD signal might reflect a superposition of transitions into a collection of four electronic states which are formed by interactions between the four electronic configurations visualized in Figure S3. We emphasize the simplicity of the figure since it takes neither excitonic coupling nor configurational interaction into account. The difference between the CD spectra of the 10 and 20 mM samples in the 300 nm region might reflect different degrees of overlap and relative orientation in the chiral J-aggregates of Fmoc groups.

### Analysis of the Temperature Dependence of UVCD Spectra

As delineated in the Results section the temperature dependence of dichroism values is complex, since data assignable to, e.g., peptide backbone, phenylalanine, and fluorene transitions exhibit different behaviors. While the temperature dependence of some dichroism data looks like a stretched sigmoidal curve, others are indicative of a multistate behavior. In order to get a somewhat quantitative grasp on these data we employed a very heuristic two-state model that we utilized in recent studies to simulate melting curves of GHG^73^ and GAG gels.^74^ The model is very simplistic in that it ignores the possibility that conformational transitions involving the peptide backbone and the Fmoc group might be correlated to some extent. We assigned different dichroism values to the states populated at low and high temperatures, respectively. The corresponding algorithm is presented in the Supporting Information. We employed this model to simulate the dichroism data at 190 nm (peptide) and 306 nm (fluorene) as obtained for 10 mM FmocFF in H_2_O and D_2_O. The results are shown in Figures S4 and S5. For both wavelengths of FmocFF in H_2_O, we could reproduce the data up to a temperature of 60 °C. The corresponding thermodynamic parameters are listed in Table S1. The respective transition temperatures, i.e., 30 and 55 °C for 190 and 306 nm. The low-temperature states are enthalpically stabilized by 120 and 70 kJ/mol, respectively. In particular, the latter value is much lower than the earlier obtained enthalpic stabilization of the gel forming GAG^74^ and GHG crystalline fibrils.^47,73^ In view of the length of the latter, this is by no means a surprising result. For FmocFF in D_2_O, the simulation of the 306 nm data again reproduces the data up to 60 °C. The simulation misses some 190 nm data between 20 and 40 °C, which seem to reflect a weakly populated intermediate. The transition temperatures are 10 (190 nm) and 30 °C (306 nm). The respective enthalpic stabilization of the low-temperature states is 180 and 40 kJ/mol. It is this relatively low enthalpy value that gives rise to the obtained stretched sigmoidal for the 306 nm dichroism.

The result of this analysis suggests a significant enthalpic stabilization of the low temperature backbone conformation in D_2_O, which however is overcompensated by an entropic stabilization of the high-temperature state, thus leading to the lowering of the transition temperature. On the contrary, the low-temperature state of fluorene in H_2_O is enthalpically more stable than the one in D_2_O, which is again compensated by a higher entropic stabilization of the high-temperature state. Overall, the resulting Gibbs free energy stabilization of the low-temperature state is less in D_2_O than it is in H_2_O for both the peptide and fluorene low-temperature state.

The above results are surprising since it is generally expected that due to the isotope effect, D_2_O would stabilize the aggregated state more than H_2_O.^47^ With regard to the backbone conformation, this notion is valid for the enthalpic contribution. However, the extent to which the aggregated state is entropically disfavored is surprising since the liquid state of D_2_O is expected to be less entropic than the one of H_2_O.^75^ In the present case, this entropy loss of the solvent must be overcompensated by very strong intermolecular hydrogen bonding in the aggregated state, as indicated by the large favorable enthalpy. Strong hydrogen bonds have a higher zero-point energy and carry a lower vibrational entropy. With regard to the fluorene moiety of the Fmoc group, the larger entropic destabilization of the aggregated state in H_2_O meets expectations. The reason for the larger enthalpic stabilization of the latter is not clear. This result seems to suggest that the hydration of the fluorene moiety carries more favorable solvation enthalpy in D_2_O than in H_2_O.

As mentioned above, the temperature dependences of the dichroism values of 20 mM FmocFF in D_2_O indicate the involvement of thermodynamic intermediates. Just to illustrate this fact, we have used the two-state model described above to simulate the respective 307 nm dichroism values between 10 and 60 °C. These data represent the transition between two intermediates populated between 10 and 30 °C and above 60 °C, respectively. A low-temperature state becomes populated below 10 °C. The two intermediate states exhibit a higher (negative) rotational strength than the low and high-temperature states. The thermodynamic parameters of the simulated transition are listed in Table S1. The 307 nm as well as the 285 nm data suggest that the aggregated state populated at high temperatures (60–70 °C) is actually the one with the highest degree of chirality.

### Comparison of CD Spectra of FmocFF in H_2_O and D_2_O

The intensity distributions of the CD spectra of 10 mM FmocFF in H_2_O and D_2_O are different. A comparison of the spectra in Figures 1 and 2 suggests the same contributions (couples and Cotton bands) to the spectrum but different relative intensities. The contributions from the peptide backbone and the phenylalanine groups are more pronounced in H_2_O while the positive Cotton bands assignable to the fluorene group are more intense in D_2_O. This observation suggests that the two chromophores are slightly differently packed with respect to each other. It is now well-known that substituting H_2_O with D_2_O may accelerate the biological process (i.e., protein aggregation) and stabilize folded and aggregated structures.^47^ With regard to short peptides, the situation is less clear. McAulay et al. investigated the influence of a H_2_O → D_2_O substitution for several blocked FF-peptides including FmocFF.^48^ At alkaline pH, small-angle X-ray scattering data suggest the formation of hollow tubelike structures with small differences between the respective radii obtained for H_2_O and D_2_O. With regard to the formation of the respective gel phases (by slowly lowering the pH), the respective moduli were found to be similar. The gelation kinetics for all FF-derivatives were found to be slower in D_2_O, contrary to what has been obtained for protein aggregation. Currently, it is difficult to compare our results with those of McAulay, since their techniques investigate FF aggregates on a scale that is larger than the ones probed by our spectroscopies. Another work that should be mentioned in this context is the investigation of a somewhat longer peptide, i.e., RFL4FR, by Hamley et al.^49^ Their UVCD data also suggest stronger hydrogen bonding between these peptides in D_2_O. Generally, the stabilization of hydrogen bonds with OD as donors due to an isotope effect results from the lower zero-point energy of the normal mode vibrations of D_2_O. Such a stabilization effect cannot be directly inferred from our data. Instead of promoting a clear upshift of the melting temperature, D_2_O seems to make the melting of Fmoc complexes and peptide sheets even less cooperative than it is in H_2_O while actually lowering the effective melting temperature. Besides revealing a more complex picture of the influence of the isotope effect on FmocFF self-assembly our data also demonstrate the necessity to obtain a complete thermodynamic picture in terms of the respective enthalpy and entropy contributions.

Our analysis thus far shows that the CD spectrum of FmocFF aggregates as formed at alkaline pH is complicated. A thorough interpretation requires more in-depth theoretical work. However, the data are indicative of FmocFF aggregates involving π–π-stacking between phenylalanine groups and Fmoc groups. The latter are sufficiently close to each other to allow for orbital overlap, while through space excitonic coupling is mainly responsible for the CD part of the phenylalanine side chains. The peptide fraction of the CD spectrum is more in line with what one would expect for a β-sheet arrangement, though differences in the region of the negative band should be noted. We now move to a discussion of IR and VCD spectra to shed more light on the latter.

### IR and VCD Spectra

On first glance, the amide I’ bands in the spectrum of 20 mM FmocFF in D_2_O seem to tell us a clear story. They are both red-shifted from their expected positions in a monomer spectrum (1648 cm^–1^ for the C-terminal pure amide I’ mode and ca. 1688 cm^–1^ for the carbonyl stretching mode of the carbamate group) by nearly 20 cm^–1^. Such a shift is generally indicative of a β-sheet formation.^76^ Here we should keep in mind that the extent of the blueshift depends predominantly on the number of incorporated strands and not on the number of residues in a strand.^77,78^ In the literature it is generally assumed that the sheets are antiparallel in nature.^39,57,79^ However, this assignment seems to be very much at odds with the obtained IR spectrum in Figure 12, which also has been observed several times before for the alkaline pregel phase as well as for the gel phase of FmocFF itself. Our physical reasoning is delineated in the following.

As shown in several studies the amide I band shift in the spectra of β-sheets is to a major extent determined by excitonic coupling between nearest neighbors in adjacent strands.^63,77,78^ As a consequence, a single linear chain of amide I oscillators already produces the obtained β-sheet spectra of amide I, if the involved modes are degenerate or nearly degenerate. For long chains, all the IR intensities are predominantly concentrated in two modes, an intense one at low wavenumbers and a weak one at high wavenumbers. The latter exists in the spectra of antiparallel and parallel in-register arrangements, but it is very weak and thus undetectable for the parallel arrangement.^78^ If, however, FmocFF dipeptides formed an antiparallel β-sheet, the degeneracy condition would not be met. While the interstrand coupling between nearest neighbors is still strong, the respective delocalization of vibrational wave functions is significantly reduced. The C-terminal and the carbamate amide I would red- and blueshift, respectively. The latter would lose intensity to the former, though not as much as in the degenerate case. That is not what we and others observe. However, if the arrangement of the strands is parallel, the main coupling occurs between the degenerate C-terminal and carbamate modes in different chains, respectively. Intensities of excitonic transition will concentrate in two equally downshifted bands, while weak upshifted bands will not be observable. That scenario would be consistent with what we observed.

The result of the above reasoning apparently contradicts the structural model that Smith et al. qualitatively derived from X-ray studies of a FmocFF gel film.^57^ According to this model, a FmocFF depicts a zipper-like structure where antiparallel β-sheets are formed by some type of heterostacking of its aromatic groups in a F-Fmoc–Fmoc-F repeat sequence. Dimers of fluorene are stuck together via J-stacking, while single phenylalanine groups are positioned between these fluorene dimers. The above sequence of aromatic groups would be qualitatively consistent with the CD couplets observed for phenylalanine transitions since respective electronic interactions between them would be through space (*vide supra*). The close proximity between fluorene groups in the dimer and their J-aggregate arrangement would, in principle, allow for the respective strong asymmetric CD signal. Hence, although the arrangements of the aromatic groups in the model of Smith et al. are at least qualitatively consistent with our data, the antiparallel backbone arrangement does not seem to be consistent with the observed amide I intensity distribution.

The above comparison assumes that fibrils in the gel and sol phases are built with sheets of similar structure. One could argue that the fibril structure in solution and in gel phases could be different. However, several lines of evidence suggest that this notion does not apply to gel phases formed in an aqueous solution. First, the micelle model of Draper and Adams suggests that worm-like aggregates, which should be predominant in our experimental conditions are the building blocks for the gel network.^5^ Second, amide I’ intensity distributions of soluble and the gel phase are very similar. Third, the FmocFF spectrum obtained with 20 mM (Figure 5) exhibits some similarity with the one of the aqueous FmocFF gel (Figure S1a), though the absence of fine structure in the latter indicates some structural differences.

In order to avoid some of the above difficulties one could invoke another model that has emerged from X-ray crystallography on FmocFF crystals formed in H_2_O-acetone mixture.^32^ Here, the peptide groups exhibit a parallel arrangement, which is in line with our IR data. However, the formation of this structure requires protonated C-termini, which form some type of carboxylate dimers. The occurrence of the band assignable to the asymmetric COO^–^ mode rules that option out. Hence, the considered structure would be subject to repulsive forces between the negative charges of the carboxylate groups. One might speculate that the carboxylate end groups could coordinate with sodium, which would serve as a neutralizing bridge between peptides. Lyszcek et al. have indeed shown that Na(I) ions can polymerize biphenyl-diacetic acid in the deprotonated state.^80^ However, this leads to a significant red-shift of the band assignable to the antisymmetric carboxylate vibration, which we did not observe (*vide supra*). Moreover, the distance of Fmoc groups in this structure (ca. 5 Å) is significantly longer than normal π–π-stacking distances (3.3–3.4 Å) and thus inconsistent with our CD data.

The only way to reconcile the model of Smith et al. with our data requires that the fibrils formed be locally crystalline in nature. As we have shown earlier for the amide I’ modes of crystalline fibrils of zwitterionic GHG and GFG peptides the internal crystal field produces a nonresonant dispersive effect that shifts intrinsic amide I modes down by 20 cm^–1^.^28,59^ This spectral change overseeds wavenumber changes caused by the excitonic coupling between amide I modes, which causes a dispersion superimposed on the above general wavenumber shift. The amide I’ wavenumbers in the spectrum of FmocFF are consistent with such a shift. Thus, there would no longer be any contradiction between the observed amide I shifts and the antiparallel structure proposed by Smith et al.

In agreement with the model of Smith, our CD and VCD data clearly suggest that the formed aggregates (worms or fibrils) are chiral in nature. Both data sets suggest a negative helical twist, the CD with regard to the phenylalanine groups and VCD regarding the twisting of the underlying β-sheet structure.^63,81^ It is well-known that helical twists of sheet structure significantly enhance the VCD signal of amide I, which would be very weak in the case of an untwisted sheet.^82^ It is reasonable to assume the same for UVCD signals. The rotational dependence of the VCD signal clearly suggests that the sample is anisotropic, which requires that the worms or fibrils are of considerable length and not statistically oriented.

To what extent is the worm model that Adams and co-workers obtained from their investigation of 2NapFF consistent with the structural insights delineated above?^79^ These authors showed that the appearance of amide I distribution similar to the one in Figure 12 coincides with the transition from spherical to worm-like micelles. With increasing concentration, these worms become rather long and bundled, which would explain the anisotropy of our sample at 20 mM. The concentration dependence of the CD spectrum indicates a heterogeneous aggregation process in which the final local order is only established in the centimolar range of peptide concentration. Interestingly, the formed structure seems to become even more compact and chiral with increasing temperature before it finally melts above 60 °C. Aggregates formed at lower concentrations start to melt earlier but the melting process is not very cooperative and stretches over a long range of temperatures.

## Summary

The study described in this paper focuses on the spectroscopic characterization of aggregates formed by FmocFF in water at very alkaline pH values which are all situated above the corresponding critical gelation value. While evidence for the aggregation of FmocFF and similar phenylalanine-based ultrashort peptides at these conditions has been reported earlier, an assessment of the structural properties of the formed aggregates and their thermal stability has not yet been accomplished. In this study, we combined UVCD, IR, VCD, and ^1^H NMR measurements which probe different structural aspects of the investigated FmocFF aggregates. UVCD spectra were found to be a complex superposition of Cotton bands and couplets assignable to peptide, phenylalanine, and fluorene electronic transitions. Most of the latter are separated from the transitions of the other two groups. Their strong dichroism reveals a helical arrangement of fluorene chromophores. A consistent interpretation of the UVCD and IR spectra suggests a crystalline antiparallel helically twisted β-sheet structure, which leads to a nondispersive redshift of amide I’ wavenumbers. Comparison of the temperature dependence of CD spectra of FmocFF in H_2_O and D_2_O reveals a complex isotope effect, where the choice of the solvent affects the thermal stability of the backbone structure and fluorene π–π-stacking differently. The UVCD spectra also show that the formation of FmocFF aggregates depends on peptide concentration in a highly nonlinear way in the range between 5 and 20 M. Our study demonstrates that the formed aggregates are highly dynamic in nature and subject to structural changes upon the addition of monomers. Overall, our study demonstrates that particularly the use of UVCD allows for the disentanglement of how the self-assembly of ultrashort peptides with an Fmoc or a similar end group affects the arrangement of the backbone, the end group, and aromatic side chains.

## Supporting Information Available

CD spectra of FF-derivatives taken from the literature, VCD spectra taken with different rotational orientations of the IR/VCD cell of the employed spectrometer, representation of electronic configurations of fluorene dimers, simulation of the temperature dependence of 10 mM FmocFF dichroism values in H_2_O, simulation of the temperature dependence of 10 mM FmocFF dichroism values in D_2_O, simulation of the temperature dependence of 20 mM FmocFF dichroism values in D_2_O (PDF)
