# Supplementary material for: Ordered Aggregates of Fmoc-Diphenylalanine at Alkaline pH as a Precursor of Fibril Formation and Peptide Gelation
Source: J Phys Chem B. 2024 Dec 23;129(1):260–72. doi: 10.1021/acs.jpcb.4c06796 (PMC11726616; doi:10.1021/acs.jpcb.4c06796)
Supplement: Supplementary file 1 — jp4c06796_si_001.pdf [file jp4c06796_si_001.pdf]

Ordered Aggregates of Fmoc-Diphenylalanine at alkaline pH as Precursor of Fibril Formation and Peptide Gelation

*Emily Hughes, Nichole S. O'Neill and Reinhard Schweitzer-Stenner**

Department of Chemistry, Drexel University, Philadelphia, PA 19104, USA

Supporting Information

*Corresponding author: rs344@drexel.edu

Temperature dependence of dichroism values

We analyzed part of the data representing the temperature dependence of dichroism values of FmocFF at different wavelength with a simple, heuristic two-state model. The simulated dichroism was calculated as follows:

$$\Delta\varepsilon_{\lambda}(T) = \frac{\Delta\varepsilon_{\lambda,lowT} + \Delta\varepsilon_{\lambda,highT} e^{-\Delta G_{hl}/RT}}{1 + e^{-\Delta G_{hl}/RT}} \qquad \text{(S1)}$$

Where $\Delta\varepsilon_{\lambda,low}$ and $\Delta\varepsilon_{\lambda,high}$ denote the dichroism values at the wavelength λ recorded at low and high temperatures, respectively. In zeroth order, the Gibbs energy difference $\Delta G_{hl}=G_h-G_l$ (*h*: high, *l*: low temperature state) is written as:

$$\Delta G_{hl} = \Delta H_{hl} - T\Delta S_{hl} \qquad \text{(S2)}$$

where ΔH and ΔS denote the respective enthalpic and entropic differences. R is the gas constant and T the absolute temperature. Here, we ignore any potential temperature dependence of the involved states' enthalpy and entropy and thus any change of the heat capacity due to the considered conformational transitions.

Table S1 Thermodynamic parameters used to simulate the temperature dependence of the dichroism at the indicated temperature

	λ=190 nm 10 mM, H_2O	λ=190 nm 10 mM, D_2O	λ=306 nm 10 mM, H_2O	λ=306 nm 10 mM, D_2O	λ=307 nm 20 mM, D_2O
$\Delta\varepsilon_{\lambda,lowT}$ ($M^{-1}cm^{-1}$)	9.0	28	5.8	19	-4.3
$\Delta\varepsilon_{\lambda,highT}$ ($M^{-1}cm^{-1}$)	1.0	0	-7.5	-2	-8.0
ΔH_{hl} (kJ/mol)	120	180	70	30	180
ΔS_{hl} (kJ/mol*K)	0.40	0.63	0.21	0.095	0.56
T_c (°C)	30	10	55	40	45

Figure S1. VCD spectrum of the amide I' region of 20 mM FmocFF in D_2O (pH 10.8) taken for different orientations of the spectrometer cell. The choice of the 0° orientation is arbitrary. The notation should be read as follows: R1: 0°, R2: 90°, R3: 180°, R4: 270°. OP1-OP4: the same orientations with the flipped cell.

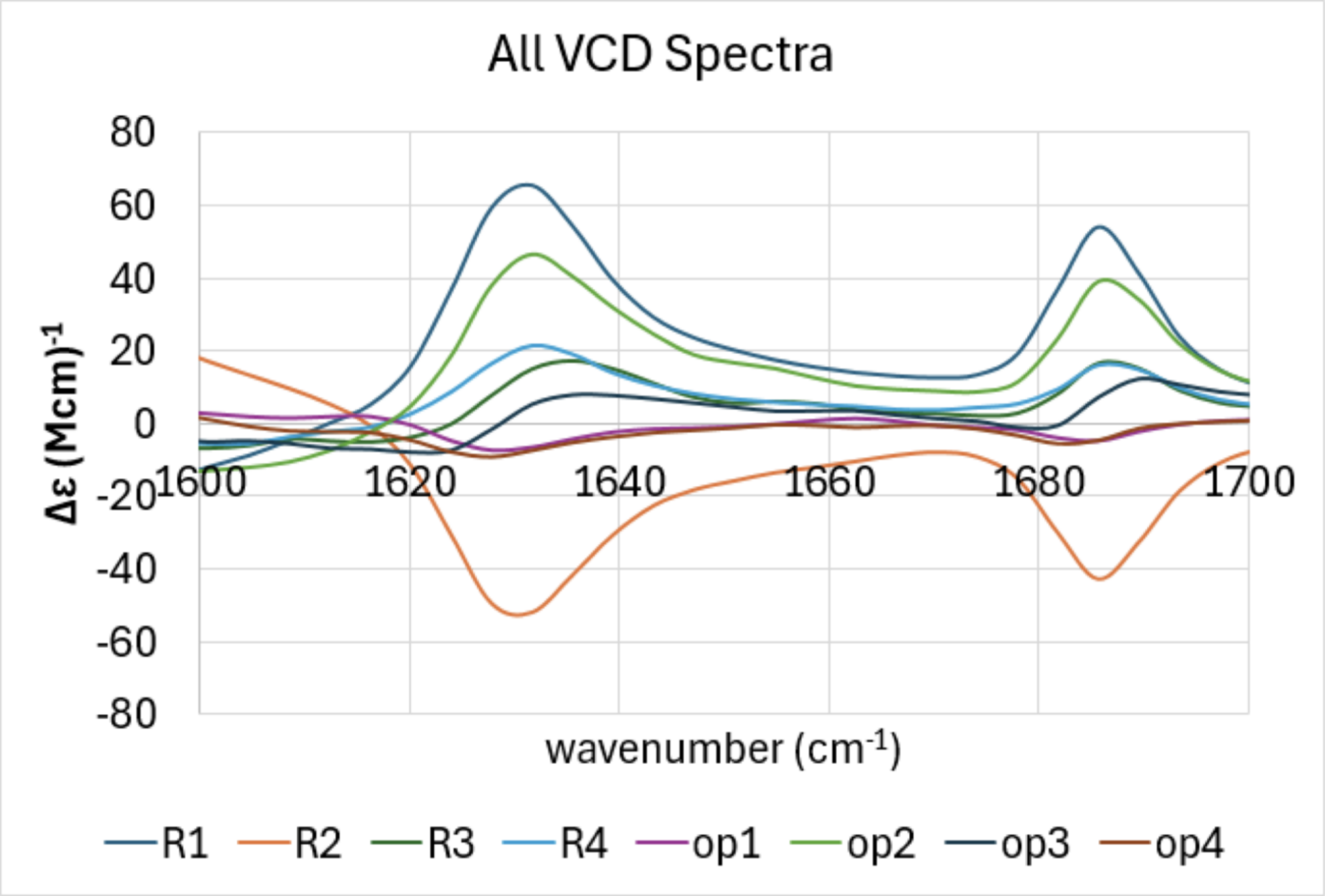

Figure S2 Left: UVCD spectra (B) of FmocF and FmocFF (A) gels formed by adding HCl to an aqueous alkaline solution. The peptide concentration was 20 mM. Taken from ref.[1] Copyright permission from Wiley & sons, 2008. Right: UVCD spectra of FmocAA gels measured at different times after the acidification of am aqueous sample. Taken from ref.[2] Copyright permission from the American Chemical Society, 2012.

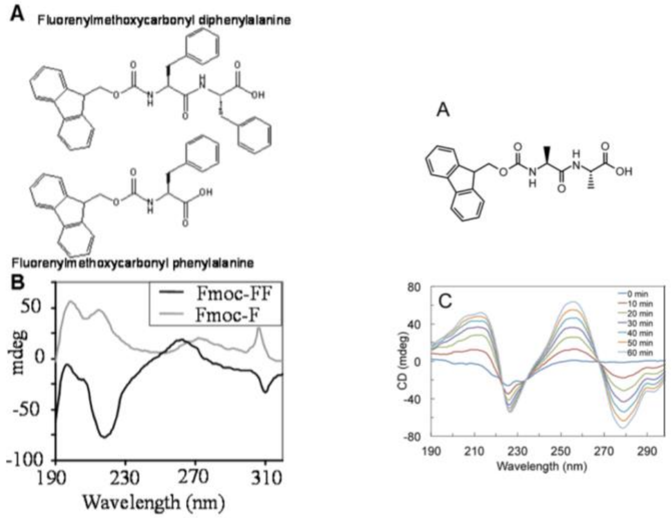

Figure S3: Possible electronic configurations of fluorene dimers with overlapping π orbitals.

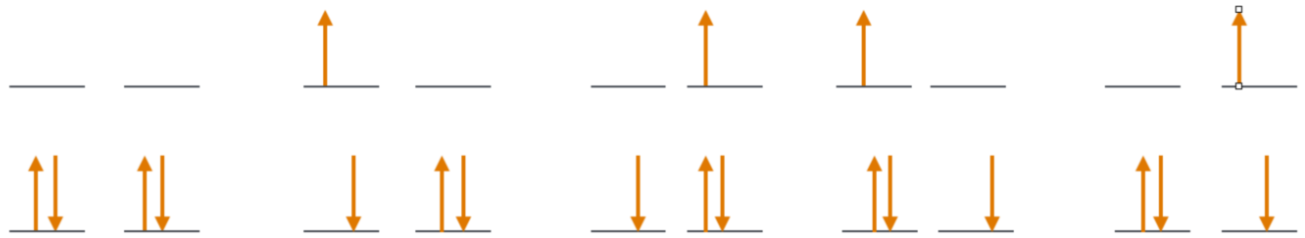

Figure S4: Simulation of the temperature dependence of the dichroism values $\Delta\varepsilon_{306}$ (*) and $\Delta\varepsilon_{190}$(+) of 10 mM FmocFF in H_2O (solid lines).

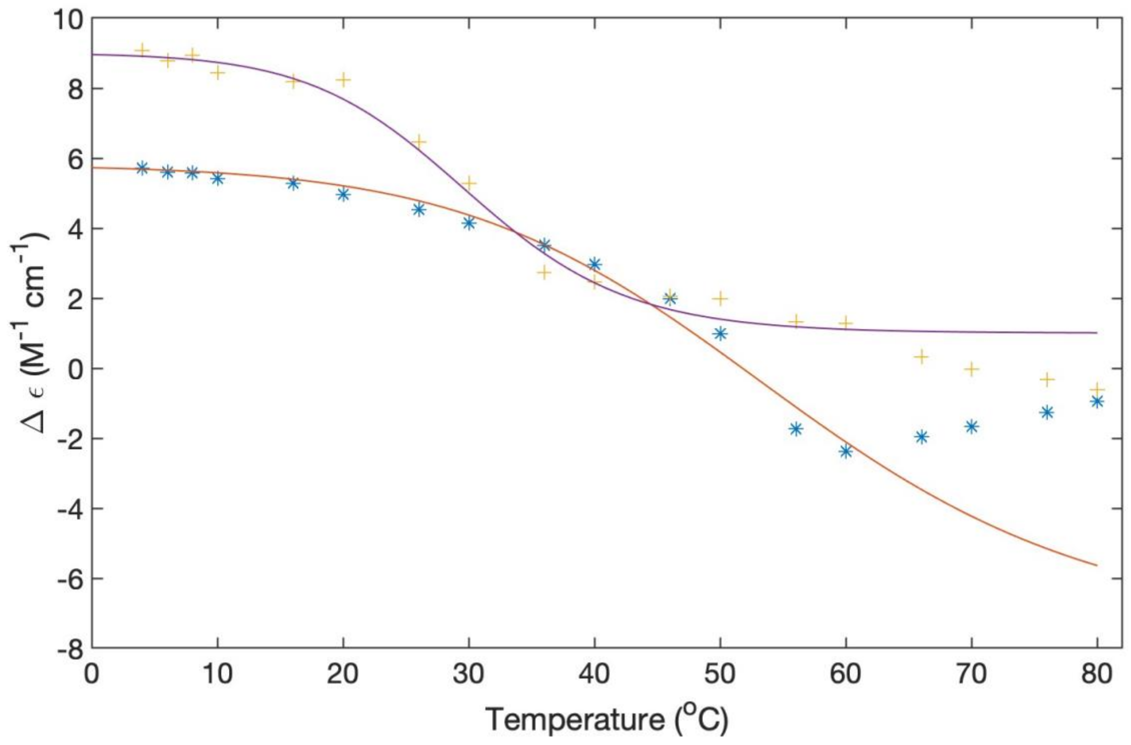

Figure S5: Simulation of the temperature dependence of the dichroism values $\Delta\varepsilon_{306}$ (*) and $\Delta\varepsilon_{190}$(+) of 10 mM FmocFF in D_2O (solid lines).

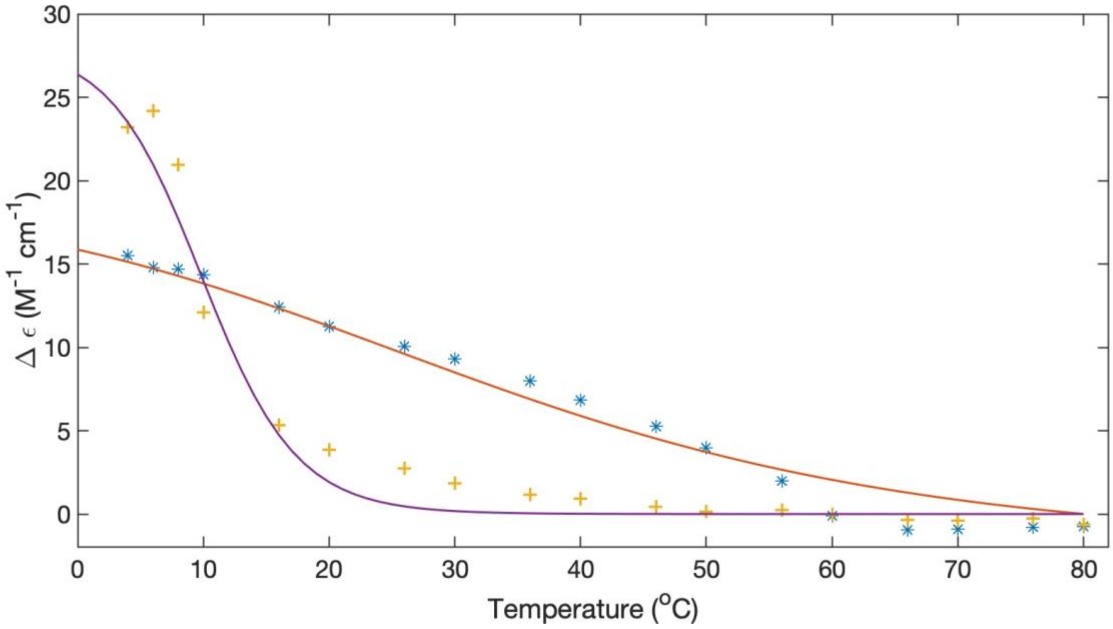

Figure S6: Simulation of the temperature dependence of the dichroism values $\Delta\varepsilon_{307}$ (*) of 20 mM FmocFF in D_2O (solid lines).

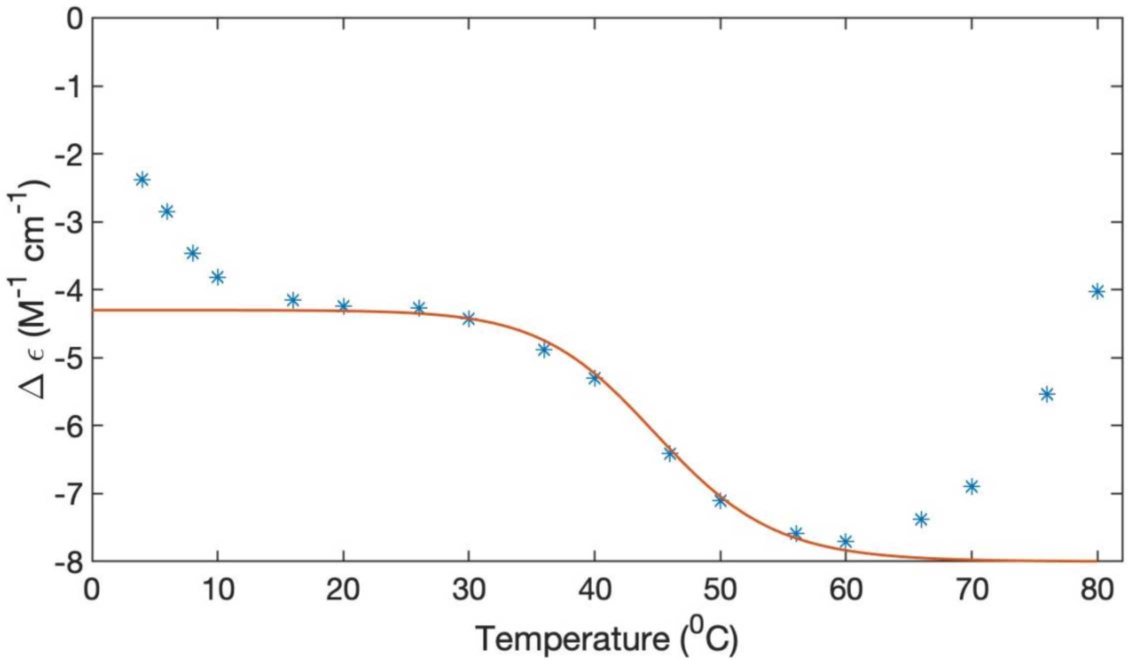

References

(1) Smith, A. M.; Williams, R. J.; Tang, C.; Coppo, P.; Collins, R. F.; Turner, M. L.; Saiani, A.; Ulijn, R. V. Fmoc-Diphenylalanine Self Assembles to a Hydrogel via a Novel Architecture Based on π-π Interlocked β-Sheets. *Advanced Materials* 2008, *20* (1), 37–41. https://doi.org/10.1002/adma.200701221.

(2) Eckes, K. M.; Mu, X.; Ruehle, M. A.; Ren, P.; Suggs, L. J. β Sheets Not Required: Combined Experimental and Computational Studies of Self-Assembly and Gelation of the Ester-Containing Analogue of an Fmoc-Dipeptide Hydrogelator. *Langmuir* 2014, *30*, 5287–5296.
